# Persistent Haglund’s disease after conventional treatments: the innovative role of radiotherapy

**DOI:** 10.1259/bjrcr.20150272

**Published:** 2016-05-05

**Authors:** Francesca di Chio, Annagrazia Cecere, Michele Troiano, Andrea Mardighian, Salvatore Parisi, Giuseppe Guglielmi

**Affiliations:** ^1^ Department of Radiology, University of Foggia, Foggia, Italy; ^2^ Department of Radiation Oncology, Scientific Institute Hospital “Casa Sollievo della Sofferenza”, San Giovanni Rotondo (FG), Italy; ^3^ Department of Radiology, Scientific Institute Hospital “Casa Sollievo della Sofferenza”, San Giovanni Rotondo (FG), Italy

## Abstract

Haglund’s disease, an inflammation of the retrocalcaneal bursa and a bone enlargement on the back of the heel, is a painful syndrome mainly caused by the exostotic prominence of the posterior calcaneus. Conventional treatment consists of rest, shoewear modification, medical therapy and, in selected cases, surgery. We report the case of a 59-year-old male with a history of severe atraumatic monolateral heel pain treated with foot orthotics, rest and surgery with partial regression of symptoms. Owing to the persistent heel pain and physical impairment after surgery, the patient underwent radiotherapy, which was successful in relieving his symptoms.

## Clinical history

A 59-year-old male complained of retrocalcaneal pain in the left foot for 1 year. The patient was obese and usually practised jogging 2 days a week for several years. On physical examination, he had pain on palpation, swelling, erythema, heat at the heel and impaired performance. The patient was unable to walk without limping. Neither abnormalities nor significant medical conditions were detected in his past history. Orthopaedic and neurological examinations were negative.

## Imaging findings and management

**Figure 1.**
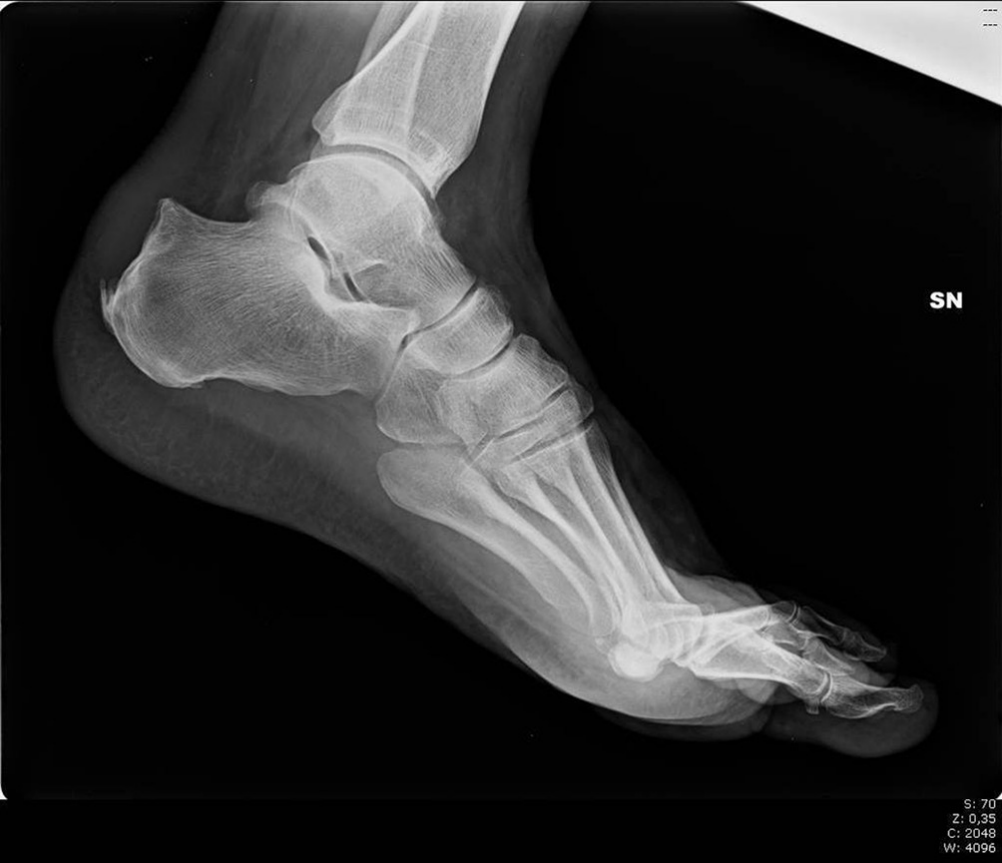
Lateral conventional radiograph of the left foot. Triangular shape of the upper rear side of the heel refers to Haglund's deformity with swelling of the distal portion of the Achilles tendon. Significantly greater phenomena of insertional enthesopathy of the Achilles tendon.

**Figure 2.**
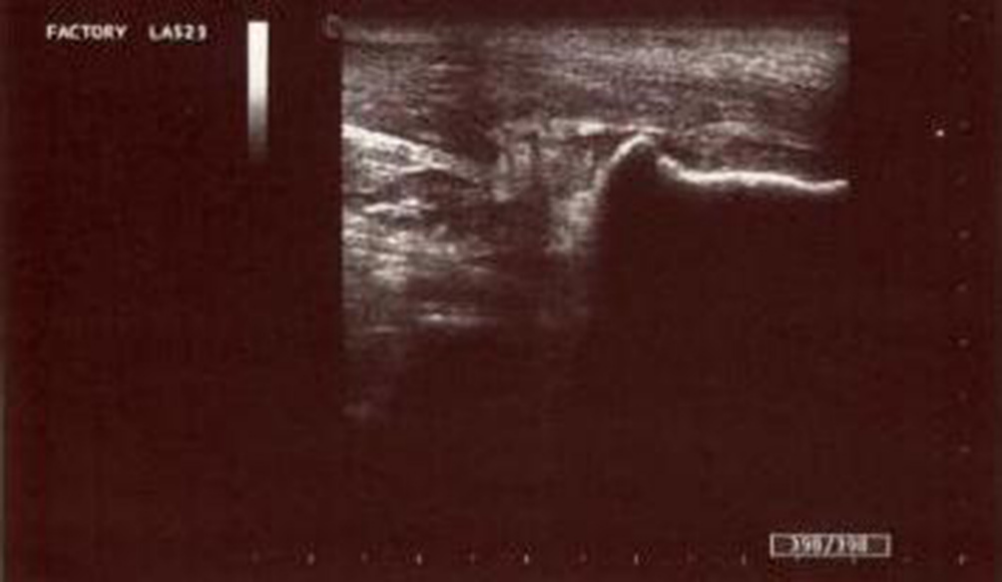
ETG of the left calcaneus and the Achilles tendon. Deformation of the superoposterior region of the left calcaneus. Calcification at the site of Achilles tendon insertion into the calcaneus. Thickening of the distal portion of the tendon with symptom of tendinosis.

**Figure 3.**
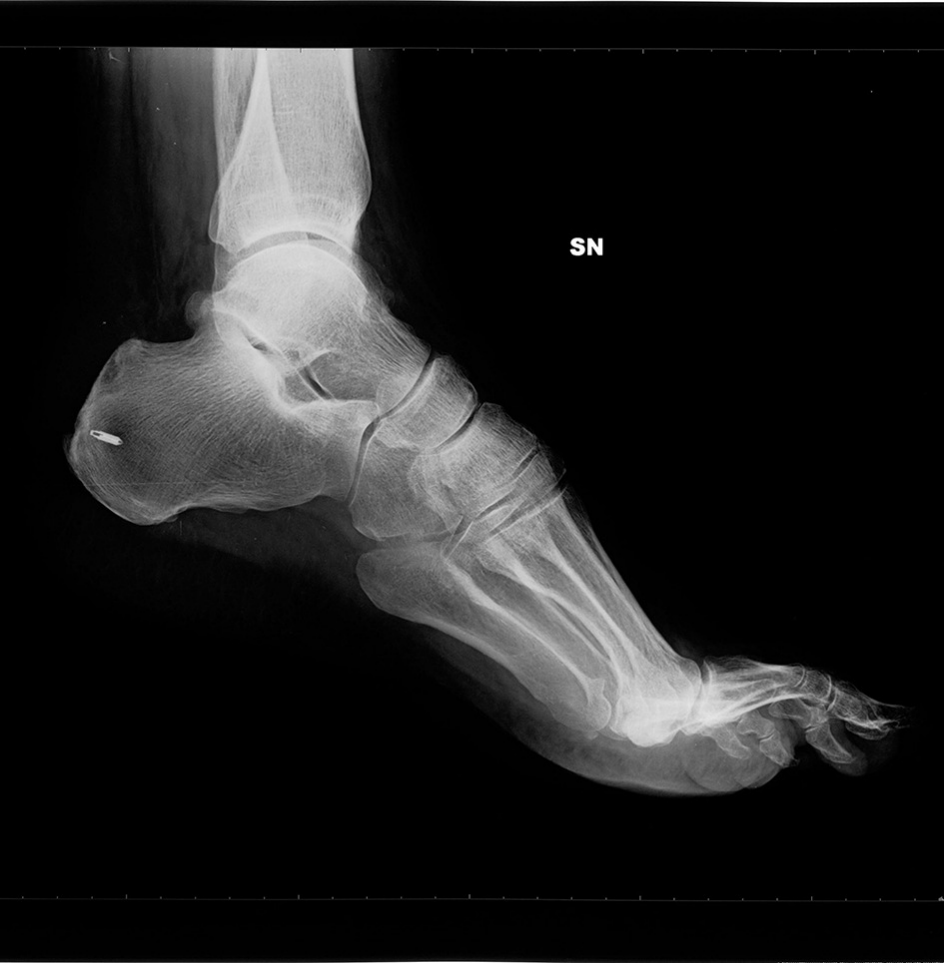
Lateral radiograph. Outcomes of surgery on the left foot with calcaneus metal screw.

**Figure 4.**
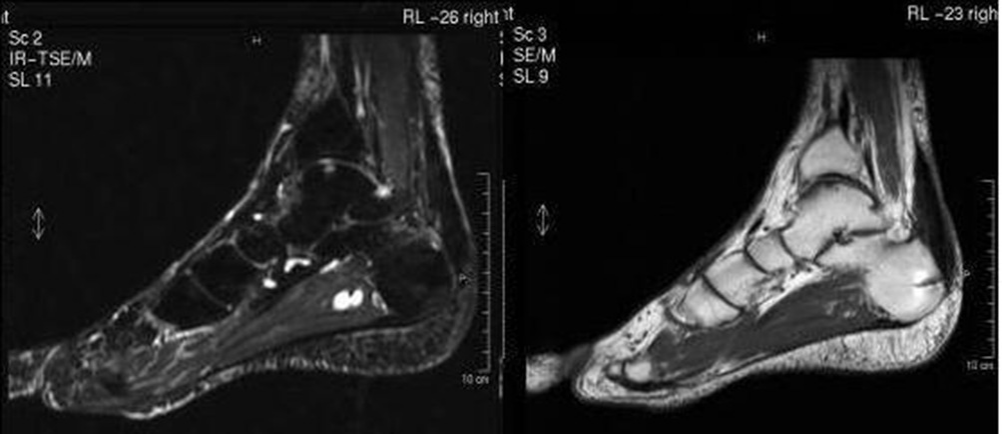
MRI of the left foot (sagittal *T*_2_ and *T*_1_ weighted images). Outcomes of tenotomy of the Achilles tendon and osteotomy of the calcaneus with metal clips and hyperaemia–oedema of the subchondral spongy bone at the site of surgery.

The clinical presentation suggested the need for a foot X-ray, followed by an ultrasound examination. A conventional radiograph of the left foot showed superolateral heel hypertrophy with enthesopathy of the Achilles tendon insertion (Figure 1). Ultrasound imaging showed calcification and thickening of the distal portion of the Achilles tendon heel insertion and calcaneal tuberosity enlargement (Figure 2). The imaging findings endorsed the diagnosis of Haglund’s syndrome and thus rest and physiotherapy were recommended. The use of foot orthotics and, if necessary, non-steroidal anti-inflammatory drugs (NSAIDs) were also recommended. After 6 months of conservative therapy, the patient came back to the hospital for surgery owing to persistent pain. In the surgical setting, the pain level was measured as 75 with a standardized questionnaire using a graphical visual analogue scale (VAS).^1^ In consideration of this medical and painful condition, surgical option was taken into account and accepted by the patient. The surgical treatment consisted of removal of the tendon conflict through an osteotomy of the calcaneus with bursectomy and tendon debridement with fixation by calcaneal axial screw. 6 months after the surgery, the patient continued to complain of pain at the calcaneal site; further radiological investigations were requested: a foot X-ray (Figure 3) and an MRI (Figure 4).

While additional information provided by the X-ray was poor, showing the osteotomy scar and the right position of the screw, the MRI revealed outcomes of tenotomy of the Achilles tendon and osteotomy of the calcaneus with metal clips and hyperaemia–oedema of the subchondral spongy bone at the site of surgery with reactive bone marrow oedema and effusion of the talar joint.

As the complex management of the patient required another treatment strategy, radiotherapy was suggested. Radiotherapy was performed on the plantar calcaneus with a single 6 Gy fraction using two oblique fields of 6 MV photon linear accelerator. Dose calculation was carried out for the isocenter.

The patient had a good response to local irradiation, with significant reduction in the pain symptomatology. The technique was considered safe and suitable. Follow-up of the patient after 24 months showed a continuous and stable pain-free period.

## Discussion

Haglund’s deformity is a syndrome caused by an exostotic prominence of the posterior calcaneus, described for the first time by the Swedish orthopaedic surgeon Patrick Haglund in 1928 as a painful hindfoot syndrome. This nosological condition is characterized by an abnormally prominent posterosuperior calcaneal process that causes posterior heel pain and is frequently associated with retrocalcaneal bursitis or insertional Achilles tendinitis.^2^

Generally, it interests 40 male patients with posterior pain, especially during sport, or younger women.^3^ Overuse and repeated stresses, in fact, may produce inflammation of the calcaneus and Achilles tendon^4^ with consequent bursitis and tendinitis. The inflammation may also affect the pre-insertional portion of the Achilles tendon^5^ for continuity.

It is necessary to examine the patient history, type and location of the pain, and finally perform an external examination of the feet^6^ in order to assess the causes of the heel pain.

Appropriate imaging is very important to make a correct diagnosis^7^ and recommend a target therapy.

A radiograph is the first imaging method to evaluate the enlarged calcaneal tuberosity and can be used as the primary examination in patients with heel pain. Lateral radiographs allow for study of the soft tissues, bone and Achilles tendon, like a stripe of soft-tissue density, with parallel margins from the soleus origin to the calcaneal insertion. Tendon calcifications, ossifications or calcaneal spurs, in fact, are clearly visible on a plain radiograph.

An ultrasound is very advantageous not only in making a dynamic examination but also in evaluating the morphology and the structure of the Achilles tendon. An MRI can be useful to assess the degree of degeneration of the Achilles tendon. Bone scintigraphy is clinically indicated in order to demonstrate an increase in osteoblastic activity, as in the case of inflammation, bone metastasis and trauma.

The first treatment of Haglund’s disease is conservative in most cases, with reduction of physical activity and rest, stretching of the gastrocnemius–soleus complex, use of ice or heat, ultrasound treatment and use of systemic and topical drugs such as NSAIDs. Foot orthotics may be useful in reducing the tension on the tendon. In the absence of significant reduction of pain after 3–6 months of conservative medical therapy,^8^ a surgical procedure may be a good choice.

**Figure 5.**
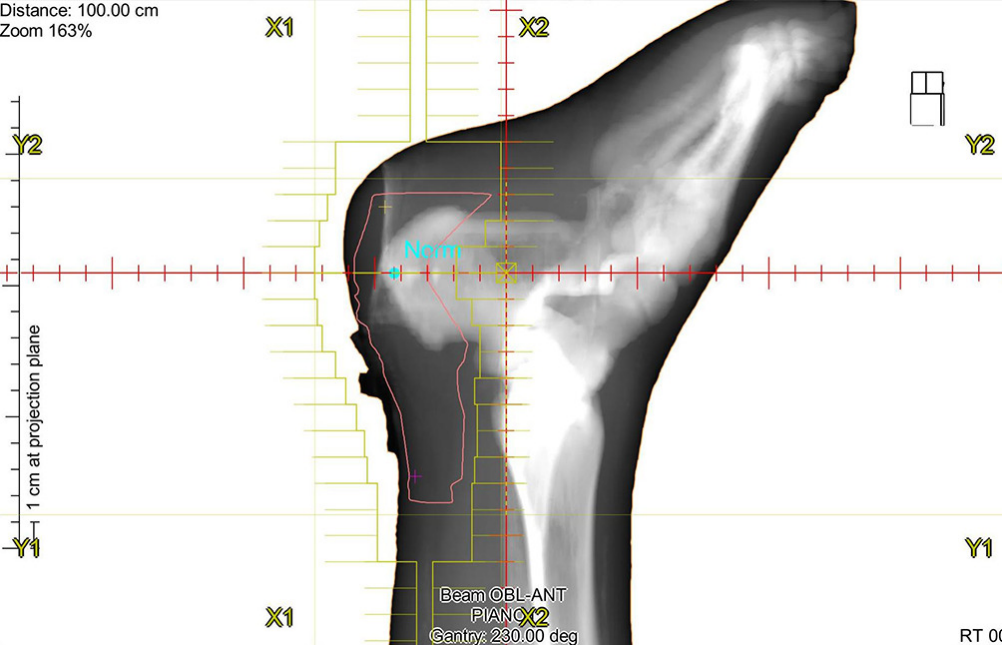
Radiotherapy. Digitally reconstructed radiograph of the oblique anterior treatment field.

**Figure 6.**
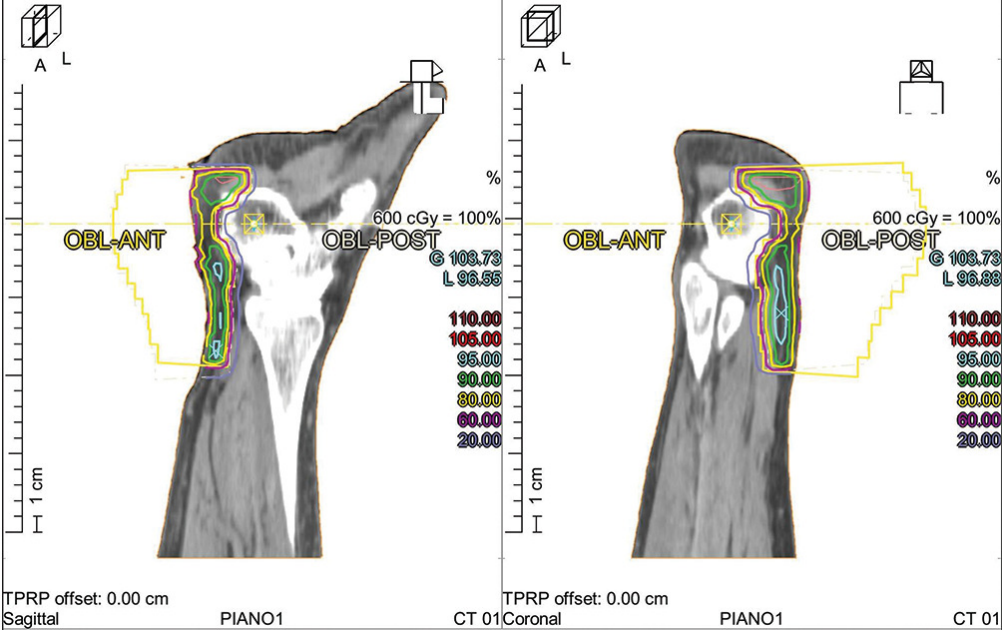
Radiotherapy. Dosimetry of treatment planning.

Although there is no scientific consensus about the gold standard surgical procedure in Haglund’s deformity, the most widely used technique consists of osteotomy of the calcaneal deformity with bursectomy and tendon debridement (Figures 5 and 6).^9,10^

Some evidences present in the literature suggest a multimodal approach in case of non-responsive Haglund’s disease. In these selected cases, the use of radiotherapy could be a great strategy for the management^11,12^ of pain control. In fact, studies by Seegenschmiedt et al^13^ and Ott et al^14–15^ confirm the benefit of radiotherapy as salvage treatment in heel pain.^13–15^

Anti-inflammatory and analgesic effects of low-dose radiotherapy (1–6 Gy) were observed in painful joint diseases, with decreased expression of cell adhesion molecules on endothelial cells,^16^ reduction of nitric oxide pathway in stimulated macrophages^17^ and reduction of oxidative burst in activated macrophages.^18^

In the literature, there is no evidence of acute and chronic side effects of radiation treatment, except for carcinogenic risk. The most important factors for radiation carcinogenesis are age at exposure and the tissues’ proliferative activity.^19^

Carcinogenic risk from radiation treatment is low because the foot and ankle are poor in red blood cells in the marrow. However, according to the effective dose concept from ICRP 60, Surenkok et al^20^ calculated a mean carcinogenesis risk factor of 1.3% for radiation portals, but no secondary cancer has been clinically observed in their study.

## Final diagnosis

Haglund’s disease was the final diagnosis.

## Learning points

1. Haglund’s disease is a painful syndrome caused by an exostotic prominence of the posterior calcaneus.
2. Conventional treatment of Haglund’s disease consists of rest, medical therapy and, in selected cases, surgery.
3. In cases of non-responsive Haglund’s disease, after medical and surgical approach, radiotherapy could represent an innovative and successful strategy.
